# Modulation of the Activity of Gold Clusters Immobilized on Functionalized Mesoporous Materials in the Oxidation of Cyclohexene via the Functional Group. The Case of Aminopropyl Moiety

**DOI:** 10.3390/molecules25235756

**Published:** 2020-12-06

**Authors:** Ana Mato, Javier Agúndez, Carlos Márquez-Álvarez, Álvaro Mayoral, Joaquín Pérez-Pariente

**Affiliations:** 1 Instituto de Catálisis y Petroleoquímica, Consejo Superior de Investigaciones Científicas (CSIC), 28049 Madrid, Spain; anamatomartinez@gmail.com (A.M.); jagundez@icp.csic.es (J.A.); c.marquez@icp.csic.es (C.M.-Á.); 2 Instituto de Nanociencia y Materiales de Aragón (INMA), Consejo Superior de Investigaciones Científicas (CSIC), Universidad de Zaragoza, 50009 Zaragoza, Spain; amayoral@unizar.es; 3 Laboratorio de Microscopías Avanzadas (LMA), Universidad de Zaragoza, 50009 Zaragoza, Spain; 4 Center for High-Resolution Electron Microscopy (CħEM), School of Physical Science and Technology, ShanghaiTech University, Shanghai 201210, China

**Keywords:** gold nanoclusters, gold atoms, cyclohexene, oxidation, XPS, SBA-15, functionalization, aminopropyl, rosemary oil, allylic oxidation

## Abstract

Gold nanoclusters and isolated gold atoms have been produced in a two-liquid phase procedure that involves a solution of gold in aqua regia and rosemary essential oil as organic layer. These gold entities have been immobilized on the ordered mesoporous silica material SBA-15 functionalized with different amounts of aminopropyl groups. The resulting materials have been characterized by XRD, N_2_ adsorption, chemical analysis, TGA, ^29^Si MAS NMR, ^13^C CP/MAS NMR, UV-vis spectroscopy, XPS, and STEM. The Au-containing materials retain the ordering and porosity of the pristine support. Gold content varies in the range of 0.07–0.7 wt% as a function of the specific immobilization conditions, while STEM evidences the presence of isolated gold atoms. XPS shows a shift of the Au 4f BE toward values lower than those of metallic gold. The catalytic activity in the oxidation of cyclohexene with molecular oxygen at atmospheric pressure parallels the Au content of the aminopropyl-SBA-15 supports. This activity is higher than that of analogous Au entities immobilized on SBA-15 functionalized with thiol or sulfonate groups, the activity decreasing in the order Au-NH_2_ > Au-SO_3_^−^ > Au-SH. This behavior has been attributed to differences in the interaction strength between the functional group and the Au entities, which is optimum for the aminopropyl groups.

## 1. Introduction

Since the catalytic properties of finely divided gold were realized [1], there has been a continued interest in the exploration of the activity pattern of gold entities comprising nanoparticles (AuNPs), with size > 2 nm, and gold clusters (d < 2 nm) as catalysts for a large variety of organic reactions, including hydrogenation and oxidations as good representative examples [2,3,4]. Different methods of preparation of gold active species have been developed along the years with the aim of rendering gold clusters and/or nanoparticles that might exhibit special catalytic properties, due to the particular electronic or geometrical features that could result from these synthesis methodologies [5,6]. An overwhelming majority of these methods lead to AuNPs, some are focused on the activity of gold clusters of well-defined geometry [7,8], while single-site approaches have gained interest more recently [9,10]. These gold entities are usually immobilized on a variety of suitable supports (mesoporous silica materials being among them [11]) to the benefit of the well-known advantages of heterogeneous catalysts: they can be easily separated from the reaction medium and eventually reused. In addition, the immobilization can also stabilize to some extent the active species and protect them from deactivation. However, the support has in general an active role in the overall catalytic reaction, because the immobilization of the active species always embraces a certain alteration of its pristine chemical state due to its unavoidable interaction with the support. Besides these chemical factors, the physical properties of the support at the nanoscale, in particular its adsorption-related properties, namely surface area, overall porosity and pore size, as well as polarity for liquid-phase reactions, can also have a notable impact.

We have recently developed a method for the easy preparation of gold clusters and AuNPs based on a procedure reported in the 18th century [12]. This consists in contacting a gold solution in a mixture of concentrated nitric acid and ammonium chloride, commonly known as aqua regia, with rosemary oil. Gently adding the oil over the aqueous gold solution, avoiding stirring, leads to the formation of a two-liquid phase system, where the oily phase remains on top of the Au-containing aqueous phase. Then, the AuCl_4_^−^ species present in the latter are spontaneously reduced at the oil-water interphase and transferred to the essential oil. The presence of finely divided gold in the oil phase of these ancient preparations was already hypothesized by authors contemporary with the invention of the recipe, but only the modern replication of the procedure has confirmed the actual existence of AuNPs together with very small gold clusters and even isolated gold atoms, though the former dominate [13]. Following this procedure but using a gold concentration in aqua regia lower than that originally reported, we have been able to immobilize gold clusters prepared according to that procedure in ordered mesoporous materials (SBA-15) functionalized with mercaptopropyl groups. The resulting materials have shown to be active catalysts for the selective oxidation of cyclohexene with molecular oxygen in liquid phase at mild conditions and atmospheric pressure [14,15]. Eucalyptus essential oil can also be used instead of rosemary oil [16] and it is worth noticing that in neither case were AuNPs found in the as-prepared, fresh catalysts. However, it has been realized that, as a function of specific preparation conditions of the gold entities, the contact of the Au-containing oil phase with the SH-bearing support required to immobilize gold may lead to the oxidation of the thiol to sulphonic groups. Then, by adjusting these conditions, it is possible to prepare Au catalysts where the Au entities are immobilized on either thiol or sulfonic groups. It has been found that if pristine SH groups are present, the catalysts are virtually inactive. Opposite to this, the catalysts where these groups were oxidized to sulfonic moieties during gold immobilization were very active for the reaction. However, it has also been found that in this last case the gold clusters initially present grow fast in the reaction medium to form AuNPs, and as a result the catalytic activity decreases notoriously with reaction time. The same phenomenon occurs if the thiol groups are oxidized to sulfonic groups prior gold immobilization [15]. However, no growth of the clusters is observed when they are immobilized on the SH groups of the inactive catalysts.

We have rationalized these experimental observations by means of the interaction strength between the Au entities and the functional groups. It is well known that the interaction between gold and SH groups is very strong, and Au-S thiolate-type bonds are formed [17,18,19]. The gold clusters would be in this way so strongly attached to the support that they are not able to participate in the reaction. On the other side, the interaction strength of sulfonate groups with gold clusters would be much weaker, and hence they would aggregate rapidly into large AuNPs. Moreover, it is also possible that the small gold clusters initially present must grow to reach the required threshold size in which they are active, as it has been proposed by Donoeva et al. [20]. They have shown that only AuNPs > 2 nm supported on amorphous silica or the all-silica ordered mesoporous material SBA-15 are active for the aerobic oxidation of cyclohexene. If this is so, the strong Au-S interaction avoids this to happen in our case, while, on the opposite side, the excessively weak Au-sulfonate interaction allows the clusters to grow fast during reaction to form large AuNPs well beyond the threshold value. In this case, as the catalytic activity of AuNPs decreases with an increase of their size [20], this excessive growing will also decrease the activity per gold atom. Following this reasoning, in order to improve the activity of Au-bearing catalysts, it would be desirable to modulate the interaction between the gold clusters and the functional group as to be in between these two extremes. On this regard, it has been reported that the interaction strength of Au with anchoring groups varies in the order Au-SH > Au-NH_2_ > Au-COOH [21]. Moreover, the binding strength of P-bearing molecules to Au is quite similar to that of SH groups, and much stronger than that of alkylamines [22]. Therefore, it can be hypothesized that the amine -NH_2_ would be the group of choice for binding Au clusters in order to improve the activity of these catalysts.

To check the validity of this hypothesis, in this work, Au clusters prepared by the aforementioned two-liquid phase procedure have been immobilized on SBA-15 mesoporous materials functionalized with different amounts of aminopropyl groups, and the resulting Au-containing samples have been tested as catalysts for the liquid phase oxidation of cyclohexene with molecular oxygen at atmospheric pressure.

## 2. Results and Discussion

### 2.1. Characterization of the Supports

**Figure 1:**
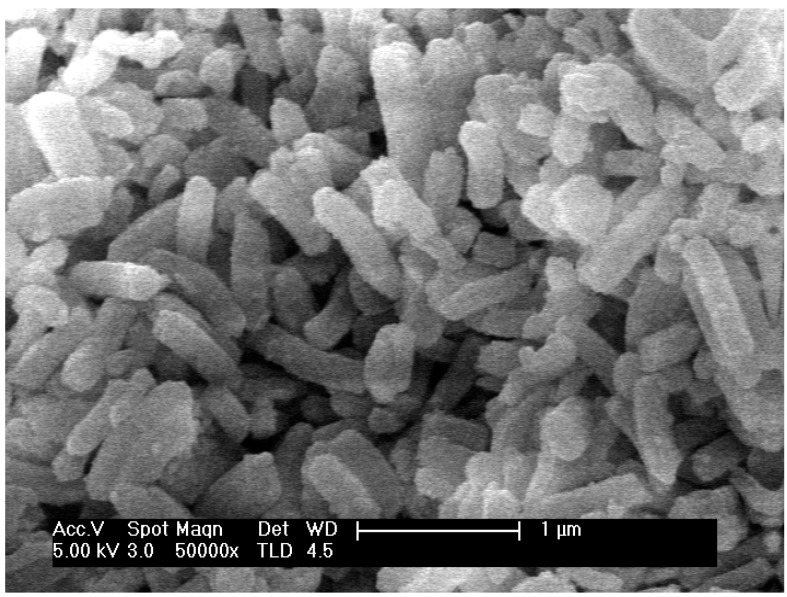
SEM of sample 0.20-E.

Following the synthesis procedure reported in the experimental section, samples with the XRD pattern characteristic of well-ordered SBA-15 mesoporous materials were achieved (Supplementary Figure S1) for the three different aminopropyl contents. In an additional essay, the molar fraction of this silane was raised to 0.30 in the synthesis gel, in an attempt to further increase the amount of amine groups in the material. This resulted in a solid with a poorly defined XRD pattern, and hence it was of no further interest. However, this helps to roughly stablish an upper limit for in-situ functionalization of SBA-15 with aminopropyl at least in the conditions prevailing in this work. The unit cell size of the well-ordered solids was quite similar, ~11.8 nm (Table 1). As evidenced by SEM analysis, they are constituted by rod-like particles, rather uniform in size and shape, being on average one micron long and nearly 0.20 microns wide (Figure 1). In this kind of morphology, the tubular mesoporous channels run all along the main particle axis.

N_2_ adsorption-desorption isotherms are presented in Supplementary Figure S2. They show a sharp increase of the adsorption branch at ~0.7 p/p_0_ relative pressure and a well-defined hysteresis loop characteristic of SBA-15 ordered mesoporous materials with uniform pore size. All of them have high surface area and pore volume (Table 1), and the latter decreases as the molar fraction of aminopropyl silane in the synthesis gel increases. Moreover, the average pore size also follows the same trend as pore volume, and decreases systematically from 8.8 nm down to 7.3 nm for the sample obtained from the gel with the highest aminopropyl content. This reduction of the free pore aperture would be consistent with the actual presence of aminopropyl moieties anchored on the pore surface. Furthermore, chemical analysis (Table 2) shows the presence of nitrogen and carbon, whose concentration in the solid follows that of the corresponding silane precursor in the synthesis gel, the nitrogen content increasing from 0.86 wt% for sample 0.06-E to 1.8 wt% for sample 0.20-E, which suggests the effective incorporation of the NH_2_-containing groups. However, the C/N molar ratio is higher than that corresponding to the propylamine backbone (C/N = 3). In this regard, it has been shown elsewhere [16] that the treatment with hot ethanol required to remove the surfactant produces at the same time the etherification of some Si-OH of the support to form Si-O-CH_2_-CH_3_.

**Figure 2:**
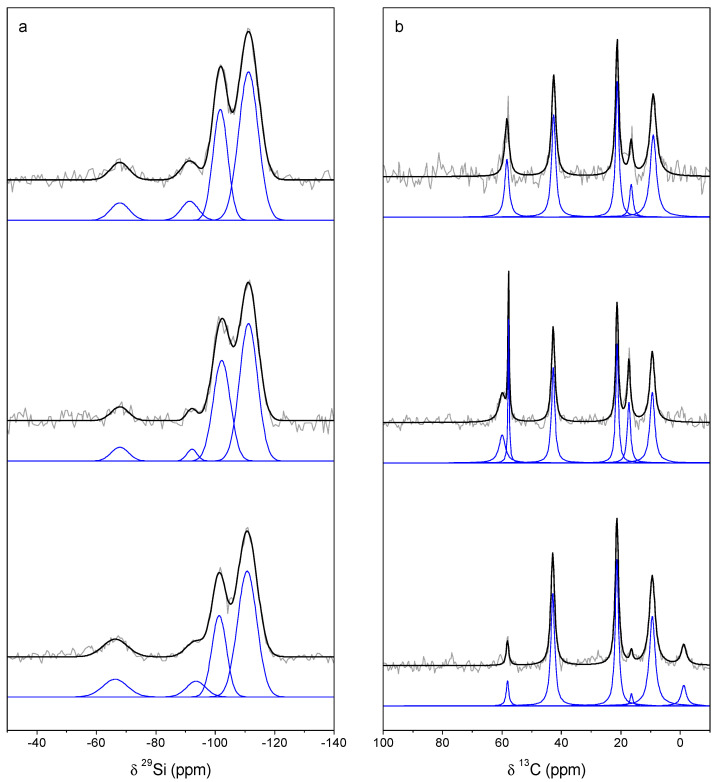
Experimental and fitted ^29^Si MAS NMR (**a**) and ^13^C CP/MAS NMR spectra (**b**) of samples 0.11-E (top), 0.11-Au-17d (middle) and 0.20-ox-Au-8d (bottom).

A direct evidence of the anchoring of n-aminopropyl moieties in the solid is provided by MAS NMR. The ^29^Si MAS NMR spectrum of sample 0.11-E is presented in Figure 2a. The three resonance signals detected at −111, −102, and −91 ppm correspond, respectively, to Si atoms in Q^4^, Q^3^ and Q^2^ environments, where Q^n^ refers to silicon atoms in coordination [Si(OSi)_n_(OH)_4-n_], where n can take the values 1, 2, 3, and 4 (Table 3). The high proportion of Q^2^ + Q^3^ sites evidences the presence in the extracted sample of a large amount of uncondensed silanol Si-OH groups. The other signal of lower intensity at −68 ppm is attributed to Si atoms in T^3^ configuration, in which they are bonded to one C atom through a C-Si silane bond, and to three oxygen atoms of the framework. The presence of this resonance signal assesses the effective condensation of 3-aminopropyl trimethoxysilane (APTMS) with tetraethyl orthosilicate (TEOS) to build the ordered SBA-15 framework. Moreover, T^3^ sites account for 5.8% of total silicon species, in very good agreement with the N/Si atomic ratio equal to 0.057 determined from chemical analysis (Table 2). The ^13^C CP/MAS NMR spectrum of this sample (Figure 2b) shows three intense signals characteristic of anchored aminopropyl moieties at 9.1, 21.2, and 42.6 ppm, corresponding respectively to the carbon atom bonded to the silicon atom, the central methylene -CH_2_- group and finally the carbon atom bonded to the terminal N [23,24]. It has been reported that the chemical shift of the methylene group of the aminopropyl backbone is sensitive to the protonation of the amine group [24], and hence the chemical shift value observed in our case would suggest that a fraction of these groups would be protonated. In addition, two signals are also observed at 16.5 ppm and 58.4 ppm, which are assigned to the methyl and methylene carbon atoms of the ethoxy groups (-O-CH_2_-CH_3_) formed by a reaction of the silanol groups with the hot ethanol used to remove the surfactant. Indeed, this surfactant removal treatment was highly efficient, as no signal at 70 ppm characteristic of the methylene carbon of the ether group of the surfactant Pluronic P123 is observed.

TG analysis of the samples (Supplementary Figure S3) shows a weight loss at temperature below 140 °C assigned to the desorption of water, while that occurring at T > 140 °C is due mainly to the desorption/decomposition of the organic species present in the samples. Several thermal events are observed in the latter case, which are centered at temperatures slightly different for the three samples: 200–300, ~350, and ~600 °C. The total weight losses at T > 140 °C (collected in Table 2) are larger than the sum of C, N, and H determined by chemical analysis (Table 2), which testifies for a contribution of the water resulting from the condensation of Si-OH and Si(OH)_2_ groups detected by NMR to the overall weight loss at high temperature.

### 2.2. Gold-Containing Samples

#### 2.2.1. Rosemary Oil Phase

**Figure 3:**
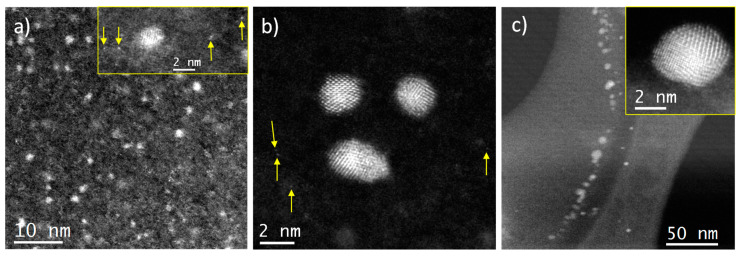
C_s_-corrected STEM-HAADF of gold entities transferred to the oil phase after one day of contact with the *aqua regia* solution (**a**,**b**). The yellow arrows indicate the existence of isolated Au atoms. (**c**) Micrograph corresponding to the sample after 8 days of contact. Inset shows a closer look of a typical nanoparticle.

As it is explained in the experimental section, the contact of the rosemary oil with the gold solution in aqua regia leads to the spontaneous reduction of gold at the interphase and the transfer of the resulting gold entities to the oil phase. This latter phase has been examined by C_s_-corrected STEM-HAADF in order to determine the nature of these metal entities, and the results have been collected in Figure 3. In the oil phase that has been in contact with the gold solution for one day, very small gold nanoclusters (Figure 3a), some of them constituted by just few atoms, are abundant, and even isolated gold atoms have been identified (Figure 3a inset, yellow arrows). Crystallized *fcc* AuNps (Figure 3b) of very small size, below 4 nm, were also observed and, although large AuNPs were detected, they were present in a minor amount. The size of these nanoparticles ranged from 1 to 16 nm, although most of them were between 1 and 4 nm, with an average size of 4.5 nm. When the contact time was extended to eight days (Figure 3c), gold clusters and single gold atoms were still visible but less abundant, while the size of the AuNPs increased, shifting the average size to 5.3 nm. Figure 3c, inset, displays the typical product when the contact time was extended, resulting in the formation of well crystallized *fcc* AuNPs. Indeed, no particles of 1-nm size were observed, and particles as large as 18 nm were detected. These observations evidence that the gold entities aggregate in the oily phase, through a slow process that takes several days. 

By comparing these results with those reported for the same two-liquid phase system in which a more concentrated solution of gold was used [13], it can be concluded that a decrease of the gold concentration in aqua regia changes the selectivity of the gold-reduction process toward the preferential formation of small gold clusters and isolated gold atoms over that of AuNPs, although the latter are still present. These results corroborate the efficiency of this simple two-liquid system to render small gold aggregates and isolated gold atoms. This suggests that the nascent gold entities initially resulting from the reduction of the AuCl_4_^−^ anions in the oil-water interphase would be protected against excessive growing by the chemical compounds present in the oil phase, which behave as efficient capping agents. It would be reasonable to think that the terpene compounds present in the rosemary oil would be responsible for this feature. However, we have observed that there is a fast change of color of the rosemary oil, which initially acquires a brown color that evolves to dark reddish brown as the contact time is prolonged (Supplementary Figure S4). This observation suggests that a reaction between the rosemary oil and the solution of gold in aqua regia takes place at their interphase. This reaction would alter the chemical composition of the rosemary oil, and work is in progress to determine the composition of the altered oily phase. 

#### 2.2.2. Au-Containing SBA-15 Materials

It can be seen in Supplementary Figure S1 that the ordering of the original SBA-15 structure as well as the unit cell size (Table 1) are nearly unaffected by the immobilization of gold. The surface area and pore volume are slightly reduced but the pore size remains unaltered (Table 1). The contact of the Au-containing oily phase with the NH_2_-functionalized materials leads to the immobilization of gold on the support. The metal content varies in the range 0.07–0.7 wt% (Table 2) in a complex manner as a function of the nitrogen content and the contact time between the gold solution and the rosemary oil. The content of gold increases with the N content of the support from 0.07 wt% for sample 0.06-Au-8d to 0.2 wt% for sample 0.11-Au-8d, and remains constant even for the sample with the highest N content (support 0.20-E). The sample with the highest Au content is the one prepared from the longest gold solution/rosemary oil contact time, 17 days. It can be noticed from the results collected in Table 2 that the immobilization process increases both the carbon and nitrogen content of the solids. The samples contain about 1 wt% more nitrogen than the original support. This is in contrast with what has been previously found for SBA-15 samples functionalized with thiol groups (which are transformed in most cases into sulfonate groups during immobilization of gold, as explained in the introduction), because in this case there is only a slight increase in carbon content upon gold immobilization, but no nitrogen is present. It is possible that the incorporation of N-containing compounds in this case is due to the basic character of the amine group, and suggests that N-bearing organic products having some acid character would be formed as a consequence of the reaction between the aqua regia and the rosemary oil that takes place at their interphase. This preliminary conclusion is important regarding the incorporation of gold, because it would mean that a fraction of the initial -NH_2_ amine groups would be protonated (-[NH_3_]^+^), losing in this way their capacity to bind gold. The ^13^C CP/MAS NMR spectrum of the sample with the highest gold content (0.11-Au-17d, 0.7 wt% Au, Figure 2b) shows the three aforementioned resonance signals characteristic of the aminopropyl moiety, together with two narrow signals at 17.3 ppm and 57.8 ppm and a less intense one at ca. 60 ppm, which are attributed to the chemical species adsorbed during gold immobilization. The ^29^Si MAS NMR of this sample (Figure 2a) is quite similar to that of the support. In this case, the T^3^ signal accounts for 5.2% of the total silicon sites (Table 3), as compared with the value of 5.8% for the support prior to gold immobilization, which suggests that a small fraction of the aminopropyl groups would have been removed during the process.

As it has been explained in the introduction, our aim in performing this study was to modulate the interaction between the gold entities and the functional group. For this reason, the sample 0.20-E was treated with ozone in the conditions described in the experimental section in an attempt to oxidize the amino group to other oxygen-bearing species, such as nitro or amide groups. The chemical analysis of the O_3_-treated sample (Table 2) reveals that the C, N, and H contents are quite similar to those of the untreated material, suggesting that little if any changes of the organic moieties were produced by this treatment. However, the amount of gold immobilized in this case is 0.6 wt%, which is substantially higher than that found for the non-pretreated material. The ^13^C CP/MAS NMR of the corresponding Au-containing sample (0.20-ox-Au-8d), in Figure 2, shows the presence of three intense signals appearing at the chemical shifts characteristic of the three carbon atoms of the aminopropyl backbone discussed above. This indicates that the amine group has not been affected by the treatment. However, a new signal characteristic of methyl silane, Si-CH_3_, appears at −1.2 ppm, which suggests the breaking of some C-C bonds of the propylamine chain. The additional signals of low intensity at 58.2 ppm and 16.4 ppm are assigned to the organic material adsorbed during Au immobilization, which is present in low amount according to chemical analysis (Table 2). In the ^29^Si MAS NMR spectrum of the sample, the four resonance signals assigned to the four different Si sites discussed above are observed. The intensity of the T^3^ environments now accounts for 10.0% of the total Si sites (Table 3), in agreement with the N/Si atom ratio equal to 0.10 of the material prior to Au immobilization (Table 2).

**Figure 4:**
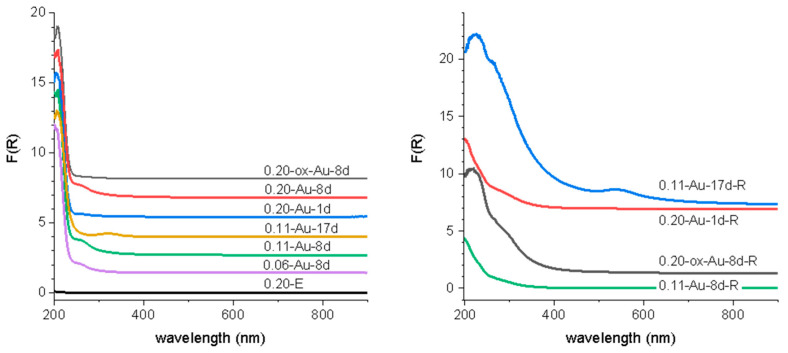
UV-Vis spectra of Au-containing SBA-15 materials and parent extracted SBA-15 (**left**) and selected spent catalysts (**right**).

The UV-vis spectra of the Au-containing materials are presented in Figure 4. For all the eight-day materials, as well as for sample 0.20-Au-1d, only a band of low intensity is observed at ~270 nm. As the contact time between the oil and water phases is prolonged to 17 days (sample 0.11-Au-17d), this band vanishes and a new one is clearly observed at ~325 nm. However, the surface plasmon resonance (SPR) band at ~520 nm characteristic of AuNPs is not observed. This certifies that no AuNPs have been incorporated in the supports, in spite that they are present in the rosemary oil solutions. This points out to a very selective immobilization of gold entities smaller than AuNPs (<2 nm). This selective uptake of small gold entities has also been described for SBA-15 functionalized with thiol groups [14,15,16]. Spectral features of the UV-vis spectra in the 250–450 nm range have been reported for small Au clusters, which are dependent upon the cluster size and charge, and the specific nature of the ligands attached to the gold atoms at the cluster surface [25,26,27,28].

**Figure 5:**
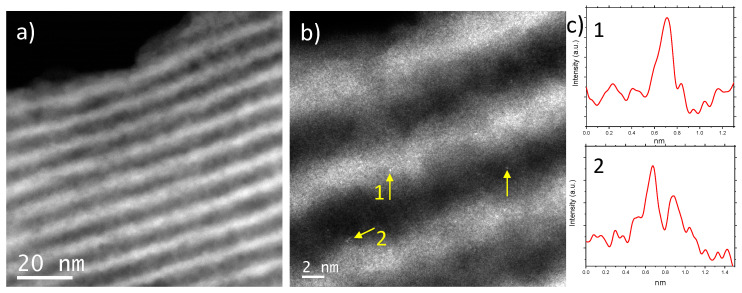
C_s_-corrected STEM-HAADF micrographs of 0.20-Au-8d. (**a**) Data recorded perpendicularly to the pore channels. (**b**) A closer look allowing the visualization of Au atoms, yellow arrows. (**c**) Intensity profiles extracted along the signals numbered as 1 and 2.

In order to further corroborate the existence of Au within the mesoporous supports and to gain information of the nature of the gold entities present in the Au-containing materials, spherical aberration corrected (Cs-corrected) STEM-HAADF studies have been carried out. The results corresponding to the Au-sample prepared at eight days of contact time (0.20-Au-8d) are depicted in Figure 5.

Figure 5a shows the image corresponding to an SBA-15 particle oriented with the pore channel system perpendicular to the electron beam. Isolated gold atoms (Figure 5b, yellow arrows) are visible in the sample, but no AuNPs are found, in agreement with the results of UV-vis spectroscopy. To further confirm that the signal observed was owed to isolated Au atoms, intensity profiles were extracted over them (numbered 1 and 2), Figure 5c, observing a peak due to the existence of a single Au atom (number 1) and due to the presence of two isolated atoms (number 2).

**Figure 6:**
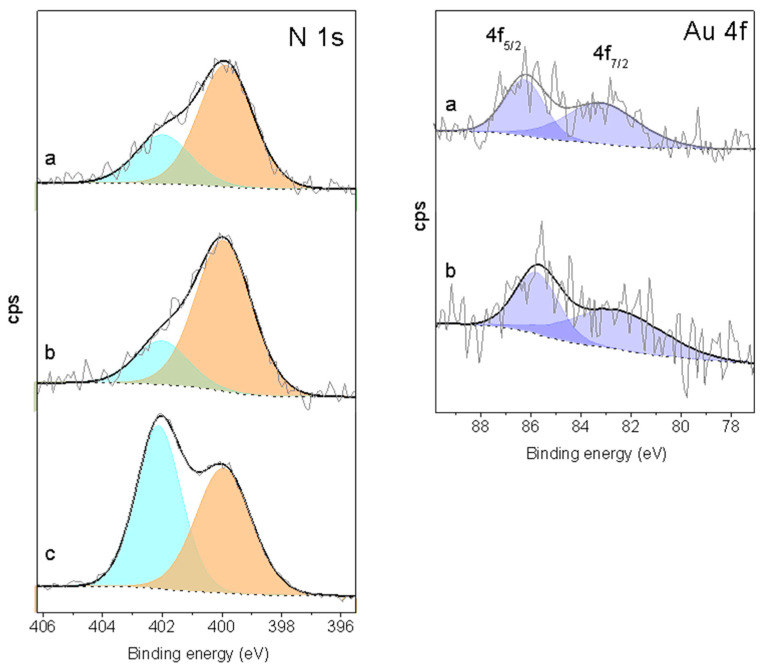
N 1s and Au 4f core-level spectra of samples (**a**) 0.20-Au-1d, (**b**) 0.20-Au-8d and (**c**) 0.20-E. Signal intensities have been normalized to a constant Si 2p peak area.

The materials have been characterized by X-ray photoelectron spectroscopy, and the results of the 0.20-E support and the two Au-samples derived from it have been collected in Figure 6 and Table 4. The Au 4f core-level spectrum of sample 0.20-Au-1d shows two broad peaks with BE of 83.1 eV for Au 4f_7/2_ and 86.2 eV for the Au 4f_5/2_ component. These two peaks are slightly shifted by ~ 0.5 eV toward lower energy for the sample 0.20-Au-8d. The BE of the two peaks show in both cases a negative shift in the range 0.9–1.5 eV respect to the value of 84.0 eV corresponding to the Au 4f_7/2_ of metallic Au foils. This shift is opposite to the positive BE shift reported for Au clusters synthesized by the same procedure here described but immobilized on SBA-15 functionalized with mercaptopropyl groups [15,16]. This result evidences that, in this case, the small Au entities are strongly sensitive to the nature of the functional groups, and in particular to the transfer of electron density between these groups and the gold entities. The causes of the negative shift of the BE observed in other systems has been discussed [29] and, although it is not yet fully understood, it has been reported for small AuNPs supported on semiconductor oxides, such as TiO_2_ and ZnO, and attributed to an increase of the electron density of the gold entities [29,30]. Furthermore, similar negative shift of the gold BE has been observed for small AuNPs supported on nanostructured SiO_2_, and attributed to a mixture of Au^0^ and Au^δ−^ states [31]. However, a contribution of geometric effects due to the large number of undercoordinated surface gold atoms present in small AuNPs has been also argued [32]. This last explanation seems to be less plausible in our case, because small gold entities are immobilized on both SH- and NH_2_-bearing SBA-15 and yet the BE shifts in opposite direction of energy. It is then quite possible that the formation of S-Au bond in the former case implies a withdrawal of electron density from the Au entities, while in the latter, nitrogen would behave as electron donor through its lone electron pair. Moreover, Au 4f level BE values smaller than that of bulk gold have been reported for Au clusters < 1.5 nm stabilized by poly(*N*-vinyl-2-pyrrolidone) (PVP) and attributed to electron donation from PVP to the Au clusters [33]. We will show below that these electronic effects strongly influence the activity and selectivity of the catalysts. On the other hand, the Au/Si atomic ratio at the surface (Table 4) is lower than that of the bulk for the 1d sample, and quite close for the 8d material.

In the N_1s_ XPS spectrum of the 0.20-E support (Figure 6) two peaks at BE 399.9 and 402.1 eV are observed. The first one is assigned to amine groups, while the one at higher BE corresponds to the positively charged nitrogen of the protonated –[NH_3_]^+^ group [23,24]. The presence of protonated N atoms in silica functionalized with aminopropyl groups have been explained by proton transfer from the silica surface silanols to the amino moiety [24]. In the case of SBA-15 functionalized by co-condensation of the silica and silane precursors as in our case and that of ref. [23], it arises most probably from the presence of the HCl acid in the synthesis gel. The atom fraction of the protonated nitrogen at the surface is 0.52, while the N/Si ratio is the same as that of the bulk (Table 4). This points to a notable homogeneity of the N distribution across the particles and hence also of the co-condensation process of the silicon and silane precursors that takes place during the synthesis of the material. Similar N_1s_ signals are visible in the spectra of the Au-containing materials, but the relative intensity of the signal of protonated N atoms decreases, being 20–30% of the total nitrogen. Moreover, the N/Si atomic ratio also decreases, showing that nearly one third of the N atoms initially present at the surface of the support were removed during gold immobilization. However, the Au/N ratio is the same for both materials, 0.013. It has been shown above that the immobilization of gold produces an increase of the content of nitrogen (and carbon as well) in the samples due to the adsorption of some organic compounds present in the rosemary oil ethanolic solution. However, no extra N_1s_ signals are observed in the spectra of the two Au-samples, which suggests that these organic compounds were efficiently whipped out from the surface by repeated ethanol washing, though the organic material occluded inside the channels was more refractory to this treatment. 

#### 2.2.3. Catalytic Activity

**Figure 7:**
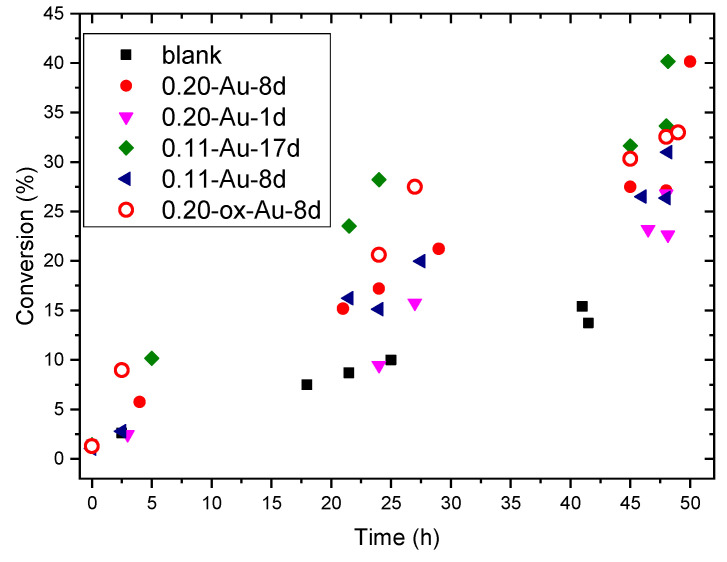
Conversion as a function of time for the liquid phase oxidation of cyclohexene with selected gold catalysts. Results of a blank test with gold-free SBA-15 are also shown for comparison.

Five selected Au-containing samples comprising different contents of gold and amino functional groups were tested as catalysts for the selective oxidation of cyclohexene with molecular oxygen at atmospheric pressure in the conditions described in the experimental section. The cyclohexene conversion as a function of the reaction time is collected in Figure 7.

**Figure 8:**
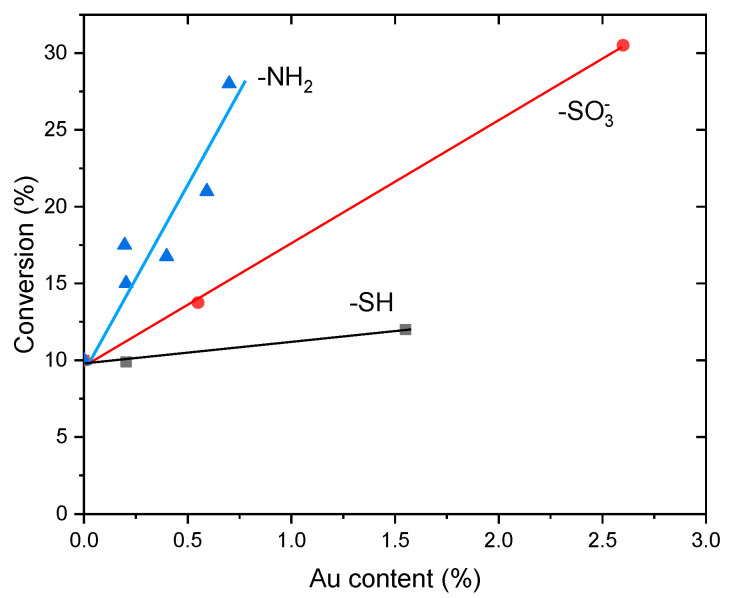
Conversion (t = 24h) vs. Au content for catalysts supported on SBA-15 silica containing anchored −NH_2_ (from this work), -SO_3_^−^ and −SH groups (taken from ref. [14]).

All the catalysts were indeed active in this reaction, and the conversion of the most active one approaches 40% after two days. However, clear differences in activity are observed, which should result from their large differences in gold content. Indeed, the conversion at 24 h of reaction time plotted in Figure 8 shows a nearly linear steady increase with the content of metal in the range 0.2–0.7 wt%. This behavior suggests that, at least within these limits, the intrinsic activity of gold is not affected by its concentration in the catalysts, and it would neither be influenced by variations of the density of aminopropyl functional groups in the range explored in this work. Once this has been established, the activity of these catalysts can be compared with those in which the Au entities synthesized in the same way here reported were immobilized on SBA-15 materials functionalized with -SH or -SO_3_^−^ moieties [14], as a function of the gold content in all cases. Plots in Figure 8 reveal that the activity of the SH-bearing catalysts is very low, hardly above that of the Au-free SBA-15 material used as blank. However, the activity of the catalysts increases notoriously if the -SH groups are replaced by sulfonic moieties. Nevertheless, the activity increases much further if gold is immobilized on -NH_2_ groups. In this case, the intrinsic activity of gold is nearly three times higher than that of the SO_3_^−^-bearing SBA-15 catalysts. Nevertheless, it should be noted that the activity per gold atom of the catalyst prepared in [20] by supporting 0.1% of gold on SBA-15 is approximately twice that of the Au/NH_2_-SBA-15 catalysts reported in this work.

**Figure 9:**
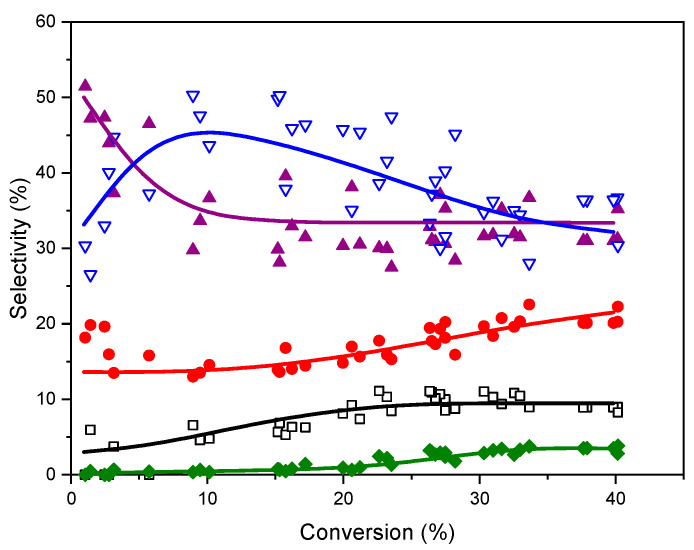
Selectivity to hydroperoxide (down triangles), cyclohexenol (circles), cyclohexenone (up triangles), epoxide (squares) and cyclohexanediol (diamonds). Data collected for the five Au catalysts of Figure 7. Lines are drawn for visual guide only and have been computed to provide a 100% value for the total sum of selectivities at any conversion level.

**Figure 10:**
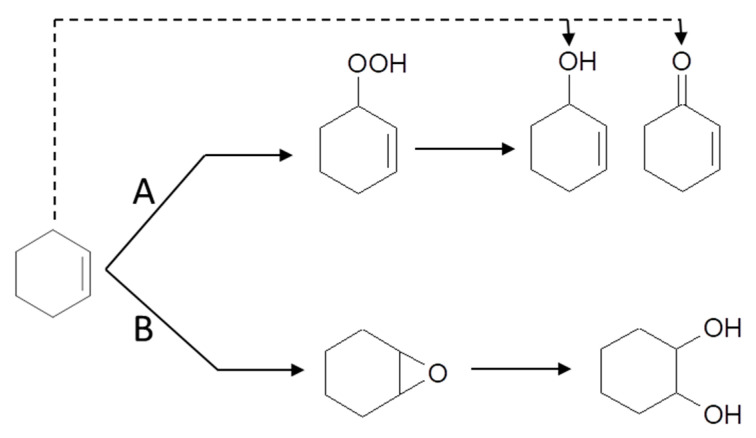
Scheme of the reaction pathway for cyclohexene oxidation under the conditions prevalent in this work.

A further clue to elucidate the reason for these differences is provided by the UV-vis spectra of the catalysts recovered after reaction. It has been shown elsewhere [14,15,16] that the gold clusters immobilized on -SO_3_^−^ groups aggregate during the reaction, and the spent catalyst showed the SPR characteristic of AuNPs. Contrary to this observation, this band is absent in the spectra of used catalysts reported in this work, except in the one with the highest Au content (0.11-Au-17d), Figure 4. Based on these observations, the activity of the catalysts and their evolution during reaction can be understood as a function of the interaction strength between the gold entities and the functional groups, as it has been hypothesized in the introduction. From this point of view, the thiol groups interact so strongly with the Au clusters that their participation in the activation of cyclohexene is hampered. On the opposite side, the Au-SO_3_^−^ interaction strength is much weaker, the gold clusters are able to be involved in the activation of cyclohexene, but at the same time they grow fast during reaction to form large AuNPs as the reaction proceeds. As it has been shown that the activity of the AuNPs decreases as their size increases [20], this will affect negatively the intrinsic activity of the gold. In the case of the -NH_2_ groups, their interaction strength with the gold entities is intermediate between these two extremes, in such a way that they can participate in the reaction but at the same time overgrowing is prevented, keeping their size below 2 nm all along the catalytic process. Further insight into the relationship between the nature of gold entities and their activity in cyclohexene oxidation can be gained by comparing our results with those reported by using well-defined triphenylphosphine-stabilized Au clusters containing 9 and 101 metal atoms with mean gold core diameter of 0.9 and 1.5 nm, respectively, deposited on SiO_2_ [20]. It has been shown that phosphine-free Au clusters with size < 2 nm are inactive in cyclohexene oxidation, and the catalytic activity appeared only after Au^0^ particles larger than 2 nm had formed. However, this conclusion does not fit well with the catalytic activity of the Au clusters immobilized on -NH_2_ groups reported here. Indeed, they are much more active than those immobilized on SO_3_^−^, but the Au entities still are <2 nm. The possibility that the isolated gold atoms and small clusters of the fresh catalysts grow to some extent during reaction cannot be excluded, but they did not certainly reach the threshold value of 2 nm reported in ref. [20]. Hence, other factors in addition to the particle size of the gold entities must be at work in the case of Au-NH_2_ configurations. They can be related to the increase of the electron density of the Au entities suggested by the XPS results. It has been proposed on this regard that the high activity in the aerobic oxidation of benzyl alcohol analogues of AuNPs in the range 2–3 nm supported on n-type semiconductors correlates positively with the increase of the electron density of the AuNPs, as evidenced by the shift of Au 4f BE toward values lower than those of the Au film [30]. It is then proposed that this increase in the electron density of metal particles facilitates the oxygen activation. However, the activation of cyclohexene has been proposed as the more probable reaction pathway in ref. [20]. Whatever the cyclohexene activation mechanism might be, it can be reasonably expected that changes in the electron density of the gold entities would affect not only the activity but also the selectivity to the different reaction products. This has been indeed observed in our case for the NH_2_-based Au catalysts as compared with those immobilized on sulphur-functionalized SBA-15 reported in ref. [14]. In order to highlight the similar selectivity pattern exhibited by the set of catalysts tested, all the individual selectivity values measured for each one of the five reaction products have been plotted in Figure 9 as a function of conversion, combining the results obtained for the five catalysts tested. It can be seen that the three products coming from the allylic oxidation of the cyclohexene ring, namely 2-cyclohexenyl hydroperoxide, 2-cyclohexen-1-ol and 2-cyclohexene-1-one account for nearly 90% of total products, the remaining 10% being due to the epoxidation of the double bond. The selectivity to cyclohexenol and its evolution with conversion is quite similar to that previously reported for sulphur-functionalized catalysts. However, noticeable differences are observed concerning the peroxide and the cyclohexenone. In the present case, the selectivity to the peroxide reaches a maximum of nearly 50% at 10% conversion, and then decreases smoothly to 35% at 40% conversion. In the case of the sulphur-catalysts, the selectivity to the peroxide at the same conversion levels is 70% and 10%, respectively. For cyclohexenone, its selectivity is about 50% at low conversion (<5%), it decreases to nearly 35% at 10% conversion, and remains virtually constant as the reaction proceeds. This is in strong contrast with what has been found for sulphur catalysts, where the selectivity steadily increases with conversion from 10% at 5% conversion, up to 60% at a conversion of 40%. As a result, the cyclohexenone/cyclohexenol ratio is 1.5% at 40% conversion, while it is 2% for the Au/S-SBA-15 catalysts [14]. This suggests that cyclohexenone is preferentially formed over the hydroperoxide at the very early stages of the reaction for the NH_2_-materials, which is just the opposite found for the S-bearing catalysts. It seems therefore that the chemical nature of the functional group mainly affects the relative selectivities of the peroxide and the enone and hardly that of the cyclohexenol. This suggests that the two first are narrowly linked one to each other in the activation pathway of the cyclohexene, and that this pathway is somehow modulated by the changes in the electronic state of the gold entities caused by the chemical nature of the functional group. The scheme in Figure 10 summarizes the reaction pathway for cyclohexene oxidation under the conditions prevalent in this work.

## 3. Materials and Methods 

### 3.1. Synthesis of Amino-Containing SBA-15 Materials

SBA-15 materials functionalized with different amounts of aminopropyl moieties were synthesized by a co-condensation hydrothermal method from gels with molar composition: 1 TEOS: x APTMS: 0.0186 P123: 6.42 HCl: 180 H_2_O, where TEOS stands for tetraethyl orthosilicate (Sigma-Aldrich, >99%, Merck KGaA, Darmstadt, Germany), APTMS for 3-aminopropyl trimethoxysilane (Sigma-Aldrich, 97%, Merck KGaA, Darmstadt, Germany), P123 for Pluronic 123, the triblock copolymer PEO_20_PPO_70_PEO_20_, m. w. ~5800 (Sigma-Aldrich, Merck KGaA, Darmstadt, Germany), and HCl for hydrochloric acid (37 wt%, Panreac Química SLU, Castellar del Vallès, Spain), and x = 0.06, 0.11 and 0.20. The synthesis procedure was as follows: 4 g of P123 were dissolved in 125 mL of 1.9 HCl contained in a 500 mL plastic bottle provided with a cover having a hole for the insertion of a PTFE stirring blade. After that, the bottle was heated at 40 °C in a silicone oil bath, and 8.2 mL of TEOS were added under gentle stirring. After 45 min, the required amount of APTMS was added (as an example, 1301 µL were added for x = 0.20) and the mixture was stirred for 21 h. After that, the mixture was poured into a 25-mL stainless-steel autoclave provided with a Teflon liner and heated statically at 100 °C for 24 h. The autoclave was then cooled down and its content filtered and washed first with distilled water and then with ethanol to finally dry it at room temperature overnight.

The dried samples were treated twice with ethanol (100 mL of ethanol per gram of sample) under stirring in a 1 L round-bottom flask at 90 °C for 24 h in order to remove the surfactant. A portion of the sample prepared from the gels with x = 0.20 was treated with ozone after the surfactant was removed. For this purpose, 0.50 g of the sample were treated for 1h at room temperature with an O_3_-rich O_2_ stream (ca. 1–2 vol% O_3_) produced by an ECO3-C3 generator (Salveco Proyectos, S.L., Coslada, Spain). The samples that were treated with hot ethanol will be denoted as x-E, where E refers to “extracted”, while “x” stands for the APTMS/TEOS ratio in the synthesis gel, i.e., 0.06-E, 0.11-E and 0.20-E. The sample 0.20-E treated with ozone will be named 0.20-ox-E.

### 3.2. Synthesis of the Gold Clusters

Gold clusters were prepared from a two-liquid phase system following a procedure previously described [13,14,15]. A gold lump of 0.3290 g (99.99%, Royston Hertfordshire, UK) was dissolved under gentle stirring in 105.2 g of aqua regia, prepared by mixing (4:1, *w*/*w*) concentrated nitric acid (65 wt%, Panreac Química SLU, Castellar del Vallès, Spain) and ammonium chloride (Sigma-Aldrich, >98%, Merck KGaA, Darmstadt, Germany), while heating at 40 °C in a sand bath. The solution contained 1:320 gold to aqua regia weight ratio. Then, 52 g of the gold solution were placed in a 100 mL decanting funnel, and 26 g of rosemary essential oil were gently poured. The oil remained as a top layer over the gold solution. The system was left at room temperature undisturbed, and aliquots of the organic layer were taken at selected intervals to prepare the Au/SBA-15 materials as described below. The chemical composition of the rosemary essential oil (supplied by El Granero Integral, Paracuellos del Jarama, Spain) was determined by GC-MS employing a gas chromatograph (Agilent 6890, Santa Clara, CA 95051, USA) coupled with a mass spectrometer (Agilent 5973N, Santa Clara, CA, USA) using a capillary column made of methylpolysiloxane (30 m × 0.25 mm × 0.25 μm), heating from 70 to 290 °C at 6 °C/min. The composition was as follows, in wt%: 24.9% 1,8-cineole, 21.9% *alpha*-pinene, 20.91% camphor, 9.06% camphene, 3.81% borneol, 3.34% verbenone, 2.59% myrcene, 2.41% *beta*-pinene, 2.01% caryophyllene, 2.00% *p*-cymene, 1.16% *alfa*-humulene, 0.98% bornyl acetate, 0.94% *gamma*-terpinene, 0.63% 4-terpineol, 0.63% *alfa*-terpineol, 0.29% *alfa*-terpinolene, 0.28% fenchone.

### 3.3. Immobilization of the Gold Clusters on SBA-15

An aliquot of 5.25 mL of the organic layer was taken after 1 day of the addition of the rosemary oil and mixed with 26.25 mL of ethanol. Then, 0.7 g of the support 0.20-E were added to this solution and the mixture was stirred at room temperature for 3h. After that, the solid was separated by centrifugation, washed with four portions of 40 mL of ethanol each and dried at room temperature. The same procedure was repeated for supports 0.06-E, 0.11-E, and 0.20-E by taking aliquots of the rosemary oil after 8 days. This contact time was also used to immobilize the gold on the support that was previously oxidized with ozone, 0.20-ox-E. In this case, 0.30 g of sample were added to a solution of 2.25 mL of the rosemary oil dissolved in 11.25 mL of ethanol. The same procedure was also applied to support 0.11-E (0.25 g) by using a 1.9 mL aliquot taken after 17 days dissolved in 9.5 mL of ethanol. The Au-containing samples will be denoted as x-Au-1d, x-Au-8d, x-Au-17d, and x-ox-Au-8d, where “x” has the same meaning as in the extracted samples, and 1d, 8d, and 17d indicate the contact time in days between the gold solution in aqua regia and the rosemary oil. 

### 3.4. Characterization Techniques

Powder X-ray diffraction analysis (Cu Kα radiation) was carried out using a X’pert Pro instrument (Malvern Panalytical, Almelo, The Netherlands). Gold content of the solid was determined by inductively coupled plasma (ICP-OES) spectrometry with an ICP PlasmaQuant PQ 9000 spectrometer (Analytik Jena, Jena, Germany). Thermogravimetric analyses were performed in a TGA7 instrument (Perkin-Elmer, Waltham, MA, USA), in air (40 mL/min) with a 20 °C/min heating ramp from 25 to 900 °C. CHNS elemental analyses were done in a CHNS-932 analyser (LECO Corporation, St Joseph, MI, USA) provided with an AD-4 scale (Perkin-Elmer, Waltham, MA, USA). Nitrogen adsorption-desorption isotherms were measured in an ASAP 2420 apparatus (Micromeritics Instrument Corporation, Norcross, GA, USA) at the temperature of liquid nitrogen (−196 °C). The samples were degassed in situ at 70 °C in vacuum for 16 h prior analysis. Surface area was determined using the BET method. The pore volume and the average pore diameter were calculated by applying the BJH protocol to the adsorption branch of the isotherm. Diffuse reflectance UV-visible (UV-vis) spectra were recorded on a Cary 5000 spectrophotometer (Varian, Victoria, Australia) equipped with an integrating sphere with the synthetic polymer Spectralon as reference. The data were expressed in absorbance units.

Scanning electron microscopy (SEM) micrographs were collected with a Philips XL30 S-FEG microscope (Thermo Fisher Scientific, Waltham, MA, USA) using Cr coating to enhance resolution. 

Transmission electron microscopy (TEM) analyses were carried out in a FEI Titan XFEG (Thermo Fisher Scientific, Waltham, MA, USA) operated at 300 kV. The microscope was equipped with a CEOS spherical aberration corrector for the electron probe to assure a maximum spatial resolution of 0.8 Å. The experiments were performed in Scanning mode using a high angle annular dark field (HAADF) detector.

Solid-state MAS NMR spectra were acquired at room temperature with an AV 400-WB spectrometer (Bruker, Billerica, MA, USA), in a 4 mm probe, using zirconia rotors with Kel-F cap. Samples were spun at 10 kHz. ^1^H to ^13^C cross-polarization (^13^C CP/MAS NMR) spectra were recorded at 400.13 MHz for ^1^H and 100.61 MHz for ^13^C, with a ^1^H excitation pulse of 2.75 μs, contact time of 3 ms, and a recycle delay of 4s. ^13^C chemical shifts relative to TMS were determined using adamantane (δ = 29.5 ppm) as secondary reference. ^29^Si MAS NMR spectra were recorded at 79.49 MHz using π/4 pulses at 55 kHz, and a recycle delay of 60 s. ^29^Si chemical shifts relative to TMS were determined using kaolin (δ = −91.2 ppm) as secondary reference.

X-ray photoelectron spectra (XPS) have been collected using a SPECS instrument (SPECS Surface Nano Analysis GmbH, Berlin, Germany) with UHV system (pressure in the range of 10^−10^ mbar), equipped with a PHOIBOS 150 9MCD energy analyzer. A non-monochromatic Mg K_α_ (1253.6 eV) X-ray source was used, working at a power of 200 W, with an acceleration voltage of 12 kV. High-resolution regions were recorded with a pass energy of 20 eV. For analysis, the powder samples were pressed into self-supporting wafers and stuck on the sample holder with double-sided adhesive conductive carbon tape. Spectra were referenced against the O 1s emission line assigned to silica (BE set to 532.9 eV [16,34,35]) to correct for charging effect. Decomposition of experimental peaks into components (70% Gaussian, 30% Lorentzian) was done using a non-linear, least squares fitting algorithm and a Shirley baseline. Relative atom ratios were calculated from the sum area of the core-level components, using the relative sensitivity factors provided by Casa XPS software (v2.3.16).

### 3.5. Catalytic Tests

The oxidation of cyclohexene with molecular oxygen was carried out by the procedure described in [14,15,16], which was adapted from that reported in ref [36]. The catalytic reaction was carried out in a 50 mL glass four-neck round-bottom flask, provided with a condenser through which water at 5 °C was circulated to minimize the evaporation of reagents and products. The flask was immersed in a silicone oil bath to keep the reaction temperature at 65 °C, measured inside the reaction mixture by a thermometer inserted in one of the necks. The experimental set-up was not protected against environmental light. The reaction mixture was made of 5.6770 g (0.0676 mol) of cyclohexene (99%, Sigma-Aldrich, Merck KGaA, Darmstadt, Germany), 0.5677 g (10 wt% referred to cyclohexene) of octane (>99%, Sigma-Aldrich, Merck KGaA, Darmstadt, Germany), 0.2839 g of a tert-butyl hydroperoxide solution (TBHP, 5 wt% referred to cyclohexene; ~5.5 M in decane, Sigma-Aldrich, Merck KGaA, Darmstadt, Germany), 4.2578 g (75 wt% referred to cyclohexene) of toluene (>99.5%, Panreac Química SLU, Castellar del Vallès, Spain), and 0.070 g of catalyst. O_2_ (1.8 mL/min) was bubbled through the stirred reaction mixture. The catalysts were previously heated at 100 °C for 1 h in the reaction flask equipped with a tube containing molecular sieve 5A in order to remove the traces of water coming from the catalysts, subsequently cooled down to the reaction temperature of 65 °C, and then the reagents were added. The trap was removed and replaced by the condenser before the reaction started. Aliquots of 0.20 mL of the reaction mixture were taken at given time intervals, filtered through a 0.45-µm PTFE filter and analysed by GC in a CP-300 instrument (Varian, Victoria, Australia), by using a FactorFour^TM^ (Varian VF-1ms) dimethylpolysiloxane capillary column of 15 m of length and 0.25 mm of i.d. Octane was used as internal standard. Five reaction products were identified, two of them resulting from the addition of oxygen to the cyclohexene double bond, namely cyclohexene epoxide and cyclohexanediol, and three coming from the allylic oxidation of the cyclohexene ring: 2-cyclohexen-1-ol, 2-cyclohexen-1-one and 2-cyclohexenyl hydroperoxide. Cyclohexene conversion was calculated from the yields of these five products.

## 4. Conclusions

Gold nanoclusters and isolated gold atoms can be efficiently produced by a two-liquid phase procedure that involves a solution of gold in aqua regia and rosemary essential oil as organic layer. The small gold entities recovered in the oil phase can be immobilized on the ordered mesoporous SBA-15 material functionalized with different amounts of aminopropyl groups. It has been found that the activity of the Au-SBA-15 materials in the oxidation of cyclohexene with molecular oxygen increases with the Au content of the catalysts. Moreover, the activity per gold atom is much higher than that of analogous Au entities immobilized on SBA-15 functionalized with either thiol or sulfonate groups, and follows the order Au-NH_2_ > Au-SO_3_^−^ > Au-SH. This catalytic performance can be rationalized based on the interaction strength of the functional groups and the gold entities, which show an optimum value for aminopropyl groups. In addition, electronic effects evidenced by the negative shift of the BE of the gold entities with respect to that of Au film seem to play an important role and would explain that gold entities <2 nm are indeed active in the reaction. Moreover, these effects also influence the selectivity to two of the three products resulting from the activation of the allylic carbon of the cyclohexene ring, the hydroperoxide and the cyclohexanone, leaving cyclohexenol unaffected. These results reveal that it is possible to modulate the activity of Au entities <2 nm immobilized on functionalized materials by a proper choice of the functional group. Moreover, the selectivity can also be influenced by the interaction between gold and these groups.

## Figures and Tables

**Table 1:** Structural and textural parameters of the supports and Au-containing samples.

Sample	Unit Cell Parameter a_0_ (nm)	S_BET_ (m^2^/g)	Pore Volume (cm^3^/g)	Pore Size (nm)
0.06-E	11.8	491	0.96	8.8
0.11-E	11.8	624	0.94	8.4
0.20-E	11.7	455	0.60	7.3
0.06-Au-8d	11.8	395	0.80	9.0
0.11-Au-8d	11.8	496	0.82	8.5
0.11-Au-17d	12.0	-	-	-
0.20-Au-1d	11.6	397	0.62	7.4
0.20-Au-8d	11.6	362	0.61	7.2
0.20-ox-Au-8d	11.6	459	0.71	7.3

**Table 2:** Chemical composition (wt%) and TG results of the supports and the Au-containing catalysts.

Sample	C	H	N	Au	Weight Loss (%) T > 140 °C
0.06-E	14.9	3.3	0.9	-	9.7
0.11-E	5.5	2.3	1.1	-	12.1
0.20-E	5.9	2.7	1.8	-	15.7
0.20-ox-E	5.8	2.6	1.8	-	16.0
0.06-Au-8d	13.2	2.9	1.9	0.07	18.7
0.11-Au-8d	11.8	2.8	1.9	0.20	18.4
0.11-Au-17d	-	-	-	0.70	12.9
0.20-Au-1d	6.6	2.6	2.6	0.40	18.2
0.20-Au-8d	11.2	3.0	2.9	0.20	21.6
0.20-ox-Au-8d	7.5	2.6	2.5	0.59	17.2

**Table 3:** Population of Si species (%) as determined by ^29^Si MAS NMR.

Sample	Q^4^	Q^3^	Q^2^	T^3^
0.11-E	56.4	31.5	6.3	5.8
0.11-Au-17d	54.4	37.7	2.7	5.2
0.20-ox-Au-8d	56.0	27.2	6.8	10.0

**Table 4:** Core-level binding energies and atomic ratios calculated from XPS analysis.

Sample	BE (eV)				Atomic Ratio			
	Si 2p	N 1s	Au 4f_7/2_	Au 4f_5/2_	N/Si	Au/Si	Au/N	N_h_/N ^a^
0.20-E	103.5	399.9 402.1	-	-	0.099	-	-	0.52
0.20-Au-1d	103.5	399.9 402.0	83.1	86.2	0.064	0.8 × 10^−3^	0.013	0.29
0.20-Au-8d	103.4	399.9 402.0	82.5	85.7	0.072	1.0 × 10^−3^	0.014	0.21

^a^ Fraction of nitrogen atoms contributing to the peak at high BE.

## Supplementary Materials

The following are available online, Figure S1: XRD patterns of representative samples, Figure S2: Representative N_2_ adsorption/desorption isotherms and pore size distributions, Figure S3: TG/DTG curves of selected supports and gold catalysts, Figure S4: Time evolution of the two liquid phase system used to produce the gold clusters.
