# Supplementary material for: Modulation of the Activity of Gold Clusters Immobilized on Functionalized Mesoporous Materials in the Oxidation of Cyclohexene via the Functional Group. The Case of Aminopropyl Moiety
Source: Molecules. 2020 Dec 6;25(23):5756. doi: 10.3390/molecules25235756 (PMC7730900; doi:10.3390/molecules25235756)

Supplementary Materials

S1. XRD patterns of representative samples

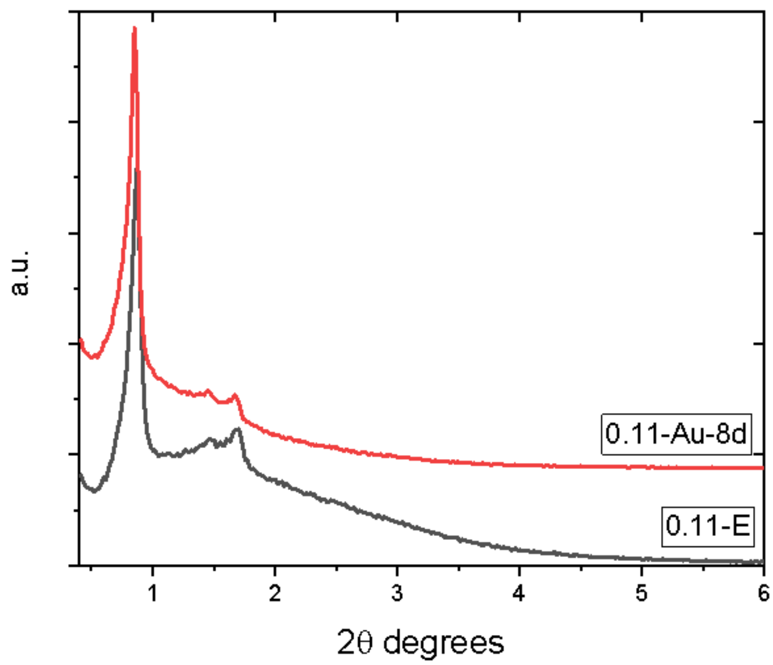

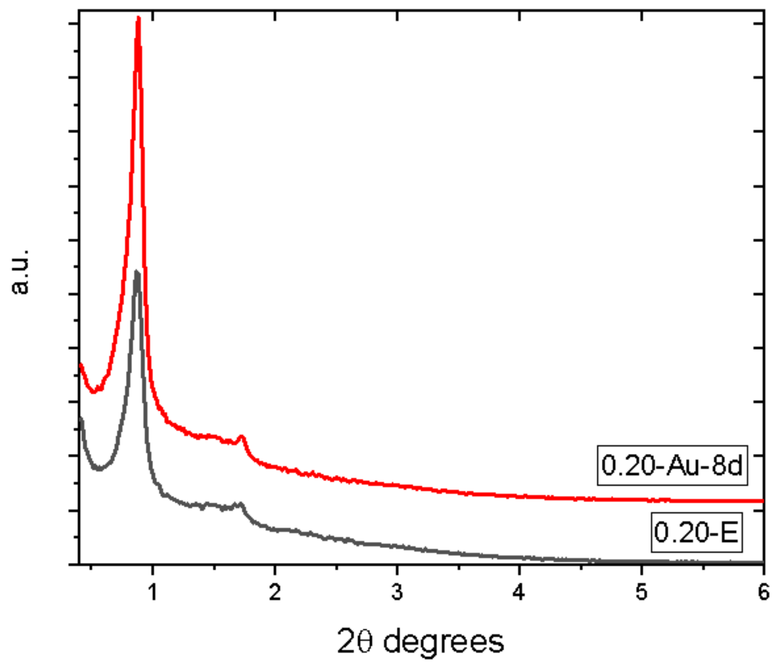

S2. N_2 adsorption/desorption isotherms of samples: 0.11-E and 0.20-E (top); 0.11-Au-8d and 0.20-Au-8d (bottom). Inset: pore size distribution.

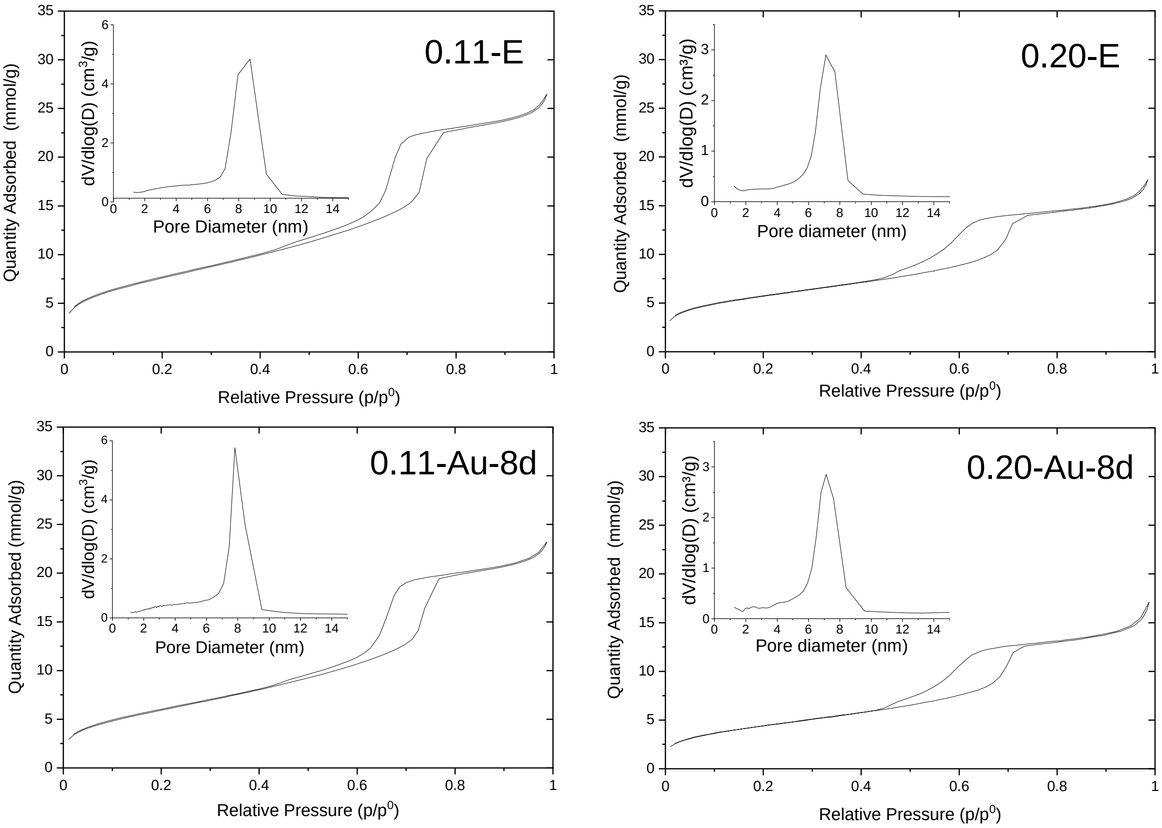

S3. TG/DTG curves of samples: 0.11-E and 0.20-E (left); 0.11-Au-8d and 0.20-Au-8d catalysts (right).

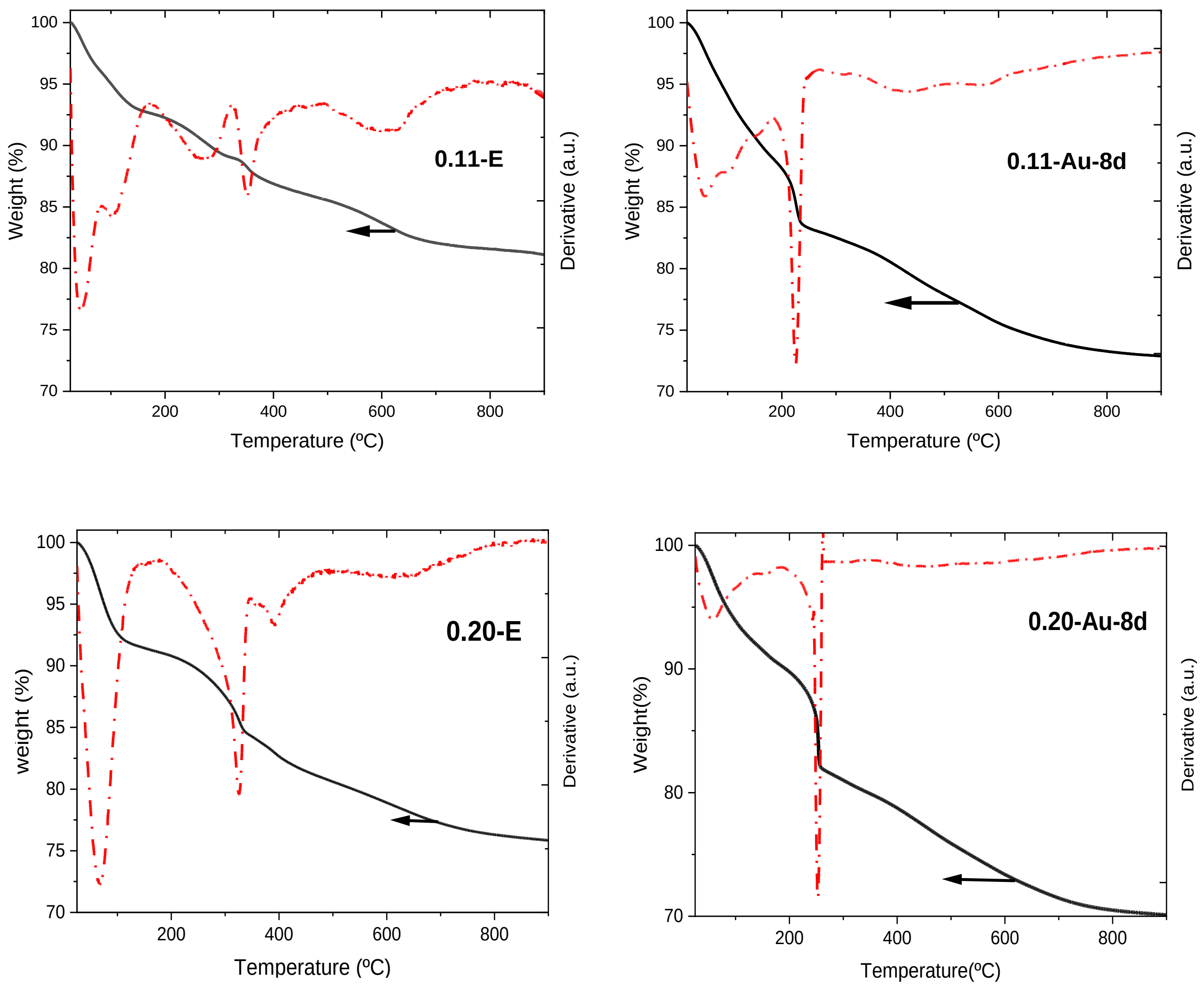

S4. Evolution of the two liquid phase system at times of a) 0 days, b) 1 day and c) 8 days.

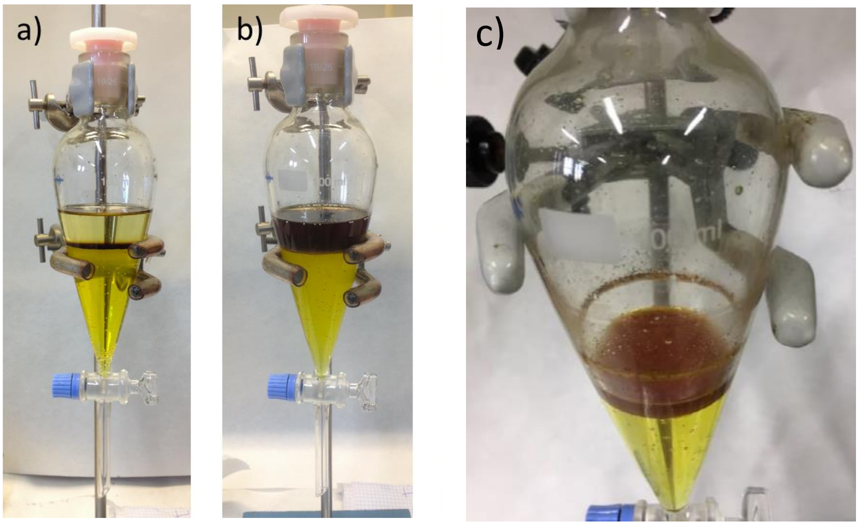

Supplement: Supplementary file 1 [file molecules-25-05756-s001.pdf]
